# Therapeutic Gene Editing of APOE4 in Sporadic Alzheimer's Disease via Prime Editor 7

**DOI:** 10.1002/advs.76658

**Published:** 2026-07-17

**Authors:** Yunkyung Kim, Gaeun Lee, Saemin An, Hanseul Park, Hongwon Kim, Jisung Kim, Soi Kang, Sumin Kim, Daeyeol Kwon, Jeonghyun Park, Yerim Hwang, Seonghun Kim, Xiuwen Yuan, Jaehoon Jeong, Hamin Lee, Hui Kwon Kim, Jongpil Kim

**Affiliations:** ^1^ Institute for Stem Cells and Regenerative Medicine (ISR) Department of Chemistry Dongguk University Seoul Republic of Korea; ^2^ Department of Integrative Biotechnology Sungkyunkwan University Suwon Republic of Korea; ^3^ College of Pharmacy & Medical Research Center Chungbuk National University Cheongju Republic of Korea; ^4^ School of Biomedical Health Science and Engineering University of Ulsan Ulsan Republic of Korea

**Keywords:** allele‐specific genome editing, alzheimer's disease, amyloid and tau pathology, apolipoprotein E4 (APOE4), patient‐derived neurons, prime editing

## Abstract

The apolipoprotein E4 (APOE4) allele is the strongest genetic risk factor for sporadic Alzheimer's disease (AD), driving Aβ accumulation, tau pathology, and synaptic dysfunction. Allele‐specific correction of APOE4 represents a promising therapeutic strategy to mitigate disease progression. In this study, we developed an APOE4‐specific prime editing strategy based on an optimized APOE4‐targeting pegRNA, enabling precise and efficient conversion of the APOE4 allele to the lower‐risk APOE3 variant. We found that PE7 targeting the APOE4 allele achieved robust and specific editing without detectable off‐target effects. This correction reduced ApoE4 protein levels and attenuated key AD‐related pathologies, including Aβ42 accumulation, tau phosphorylation, and activation of the ERK1/2 pathway in APP/APOE4 knock‐in (KI) mice. Notably, PE7 treatment enhanced neuronal survival and improved cognitive performance in these mice. Furthermore, in human induced neurons derived from APOE3/4 heterozygous AD patient fibroblasts, PE7 consistently corrected the APOE4 allele and suppressed both amyloid‐ and tau‐associated pathologies. These findings establish PE7‐mediated APOE4 correction as a precise and efficient therapeutic genome‐editing strategy with translational potential for sporadic AD.

## Graphical abstract

Prime Editor 7‐mediated conversion of APOE4 to APOE3 alleviates Alzheimer's disease‐associated pathology in AD mouse models and patient‐derived neurons and improves cognitive performance in vivo, supporting therapeutic genome editing as a promising strategy for APOE4‐associated neurodegeneration.

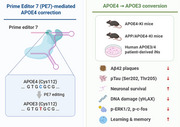

## 1 Introduction

Alzheimer's disease (AD) is the most common form of dementia and a progressive neurodegenerative disorder characterized by memory loss, cognitive decline, and accumulation of amyloid‐β (Aβ) plaques and neurofibrillary tangles in the brain [1]. While the majority of AD cases are sporadic and occur later in life, genetic risk factors play a critical role in disease susceptibility and progression [2]. Among the known genetic risk factors, the apolipoprotein E (APOE) gene, particularly the APOE4 allele, is the strongest genetic contributor to late‐onset sporadic AD [3]. There are three common allelic variants— APOE2 (Cys112, Cys158), APOE3 (Cys112, Arg158), and APOE4 (Arg112, Arg158)—with approximate global frequencies of 8%, 78%, and 14%, respectively [4]. While ApoE3 is considered the neutral isoform and ApoE2 may confer protection, ApoE4 is associated with impaired Aβ clearance, increased Aβ aggregation, heightened neuroinflammation, altered neuronal function, and reduced synaptic plasticity [5]. Consequently, individuals carrying one copy of the APOE4 allele have an approximately 2–4‐fold increased risk of developing AD, and those with two copies face an 8–16‐fold higher risk compared to non‐carriers [6]. Notably, approximately 20–25% of the general population carries one APOE4 allele, and 40–65% of individuals with AD carry at least one copy [7]. Thus, correcting the APOE4 allele emerges as a critical therapeutic strategy with the potential to mitigate disease susceptibility and modify the course of Alzheimer's disease, highlighting the importance of precise genome‐editing approaches.

Prime editing (PE) is a precise genome‐editing platform that enables programmable base substitutions, small insertions, and deletions without introducing double‐strand breaks or requiring donor DNA templates [8]. By combining a Cas9 nickase with an engineered reverse transcriptase and a prime editing guide RNA (pegRNA), PE enables highly versatile sequence rewriting with reduced bystander effects compared with earlier editing platforms. Among the optimized PE variants, PE7 incorporates the RNA‐binding N‐terminal domain of the La protein to enhance pegRNA stability and improve editing efficiency across diverse cellular contexts [9]. These advances have expanded the applicability of prime editing to in vivo therapeutic settings, including disease‐relevant models of the central nervous system, highlighting its promise as a precise genome‐editing strategy for neurological disorders. Prior to the advent of PE, earlier genome editing tools such as Cas9 nucleases and base editors have been employed to investigate or modify AD‐related genes. For instance, CRISPR‐Cas9 has been utilized to achieve in vivo editing of Bace1 in adult mouse neurons, attenuating amyloid‑β accumulation and ameliorating AD‐like cognitive impairments in AD models [10]. In another example, a base editing strategy was applied to introduce the protective A673T mutation into the APP gene in vitro, thereby preventing amyloid‐β aggregation and highlighting the therapeutic potential of base editing in AD [11]. However, despite these advances, no prior studies have demonstrated allele‐specific correction of the APOE4 variant that leads to observable therapeutic benefits in AD models. Thus, in the present study, we aimed to establish an APOE4‐specific prime editing strategy—based on an optimized APOE4‐targeting pegRNA combined with the PE7 system—to enable efficient conversion of the APOE4 allele to APOE3 and to evaluate whether this approach could provide therapeutic benefit for AD.

We demonstrate efficient editing of the APOE4 allele through delivery of PE7 in NIH3T3 cells and APOE4‐expressing neurons. Furthermore, PE7‐mediated targeting of APOE4 significantly reduced AD‐related molecular phenotypes, including Aβ42 production, and improved cognitive function in an AD mouse model carrying APOE4. Finally, in human induced neurons (iNs) derived from APOE3/4 patient fibroblasts, PE7 successfully corrected the APOE4 allele and mitigated AD‐related molecular phenotypes. These findings suggest that PE7 targeting APOE4 represents a promising in vivo gene editing strategy for the treatment of APOE4‐mediated sporadic AD, with potential to slow or prevent ApoE4‐driven disease progression.

## 2 Results

### 2.1 Preparation of Prime Editor 7 for Conversion of APOE4 to APOE3

The apolipoprotein E gene variant APOE4, which encodes arginine residues at amino acid position 112, is the largest and most common genetic risk factor for late‐onset Alzheimer's disease [12]. To improve the efficiency of intended edits at the APOE4 allele, we employed DeepPrime [13], a machine learning‐based prediction tool for assessing pegRNA activity. Using this approach, we identified six guide sequences with high predicted editing efficiency at the APOE4 allele (Figure S1a). For each guide sequence, multiple pegRNAs were prepared with various PBS and RTT lengths to maximize editing performance (Figure S1a, upper panel; Table S1). Moreover, to enhance prime editing efficiency by modifying PAM sequences or adjacent sequences near the intended edit, we generated additional pegRNAs introducing silent mutations (Figure S1a, lower panel). In addition, to prevent degradation of the 3’ extension, the tmpknot motif was appended to all pegRNAs.

**FIGURE 1:**
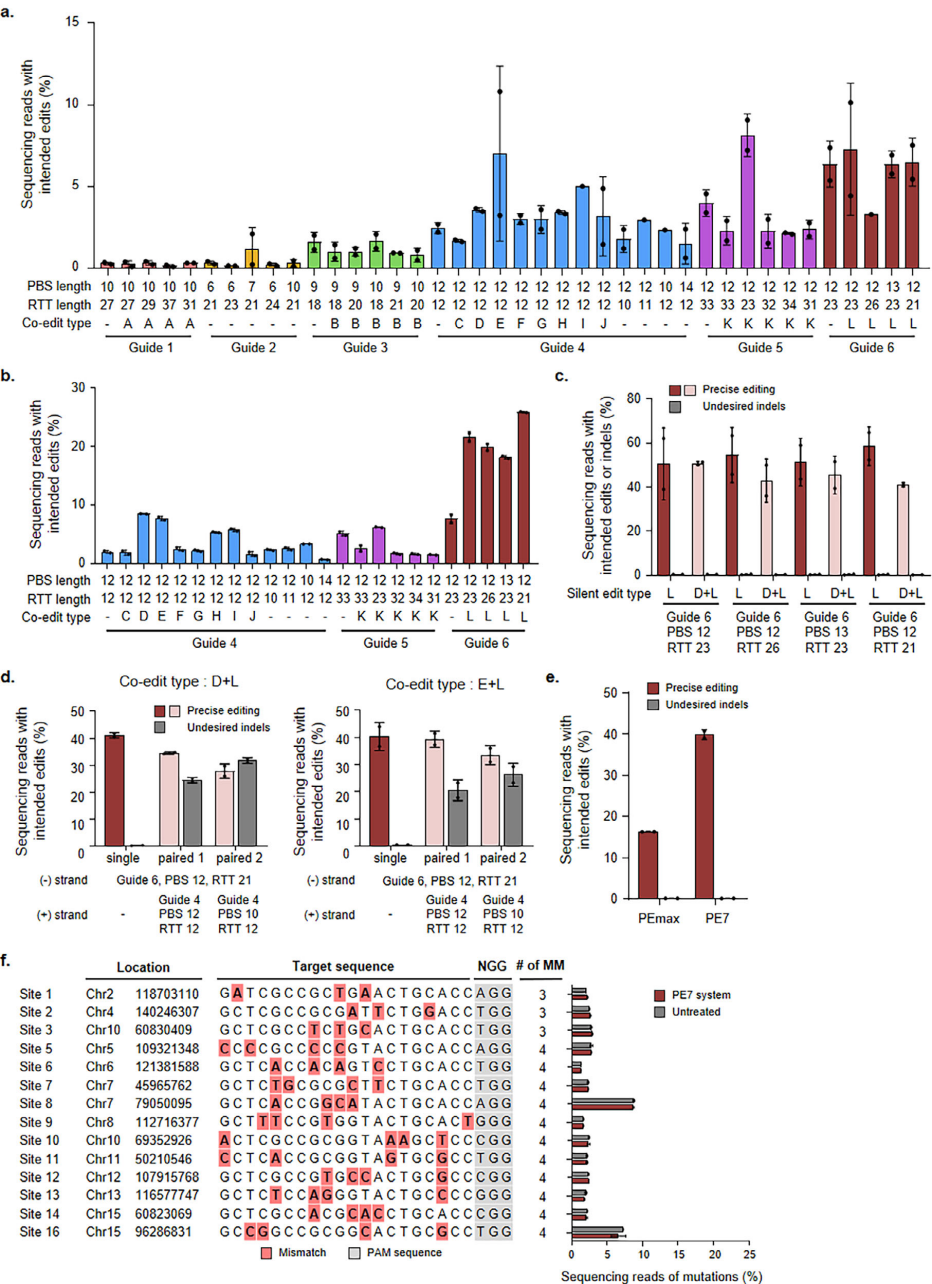
Selection of optimal pegRNA for therapeutic correction of the APOE4 c.334C>T. (a, b) Targeted deep sequencing results for 40 pegRNA candidates in mouse fibroblasts (a) and 24 pegRNA candidates in NIH3T3 cells (b). (c‐e) Prime editing efficiencies measured in NIH3T3 cells using various editing strategies, including co‐edit type combinations (c), dual pegRNA delivery (d), and the use of PE7 system (e). (f) Off‐target prime editing evaluation of the selected pegRNA with PE7 system in NIH3T3 cells. Left: sequence information for potential off‐target sites in the mouse genome. Right: targeted deep sequencing results at these sites. Mutations comprised insertions, deletions, and substitutions. (a‐f) Error bars represent the standard deviation of two replicates.

To evaluate the editing efficiencies of pegRNAs, mouse fibroblasts carrying the APOE c.334C>T mutation were transduced with PEmax lentiviruses and selected with blasticidin. Subsequent delivery of pegRNA‐encoding lentiviruses, followed by puromycin selection, yielded enriched populations of transduced cells. Seven days after transduction, editing outcomes of all designed pegRNAs were quantified by deep sequencing. Among them, 24 pegRNAs derived from guides 4, 5, and 6 exhibited consistently high efficiencies (Figure 1a). When these pegRNAs were further validated in NIH3T3 cells, the pegRNA with guide 6 (PBS 12, RTT 21, co‐edit type L) showed the highest efficiency, achieving 25.9% editing (Figure 1b).

Previous studies have reported that incorporating co‐edit types, particularly synonymous (silent) substitutions, can enhance prime editing efficiencies by facilitating mismatch repair evasion and stabilizing the edited product [14]. We then assessed the effects of co‐edit type incorporation by measuring the editing efficiencies of pegRNAs in which the co‐edit types introduced various silent mutations alongside the intended c.334C>T edit. Notably, for a pegRNA with guide 4 (PBS 12, RTT 12), we found that co‐introduction of c.333G>C (co‐edit type D) or c.333G>T (co‐edit type E) increased the editing efficiency by approximately 4.2‐fold and 3.8‐fold, respectively (Figure 1b). Based on these findings, we incorporated co‐edit types L and D in four pegRNAs derived from guide sequence 6; however, combining these co‐edit types did not yield additional improvements in editing efficiency (Figure 1c). To further optimize prime editing, we implemented the strategy of simultaneously delivering two pegRNAs targeting both the top and bottom strands. However, we found that co‐delivery of pegRNA pairs resulted in a significant increase in unwanted indel formation compared with the single‐pegRNA group (Figure 1d). Next, we adopted PE7, which improves prime editing efficiency by preventing pegRNA degradation [9]. The PE7 system exhibited a 2.4‐fold improvement in editing efficiency compared with the PEmax system (Figure 1e). Therefore, we selected the PE7 system along with a pegRNA with guide 6 (PBS 12, RTT 21, co‐edit type L) for subsequent experiments (Figure S1a,b).

Finally, we evaluated off‐target prime editing activity in NIH3T3 cells treated with the PE7 system using targeted deep sequencing. A total of 16 potential off‐target sites with up to four mismatches relative to the guide sequence were identified using Cas‐OFFinder [15], although sites 4 and 15 were excluded from analysis owing to sequence complexity and unsuccessful PCR amplification in NIH3T3 cells. Deep sequencing revealed no detectable off‐target prime editing at any of these sites, confirming the high specificity and safety of our optimized prime editing system (Figure 1f).

### 2.2 Precise and Efficient Conversion of APOE4 to APOE3 via Prime Editor 7

**FIGURE 2:**
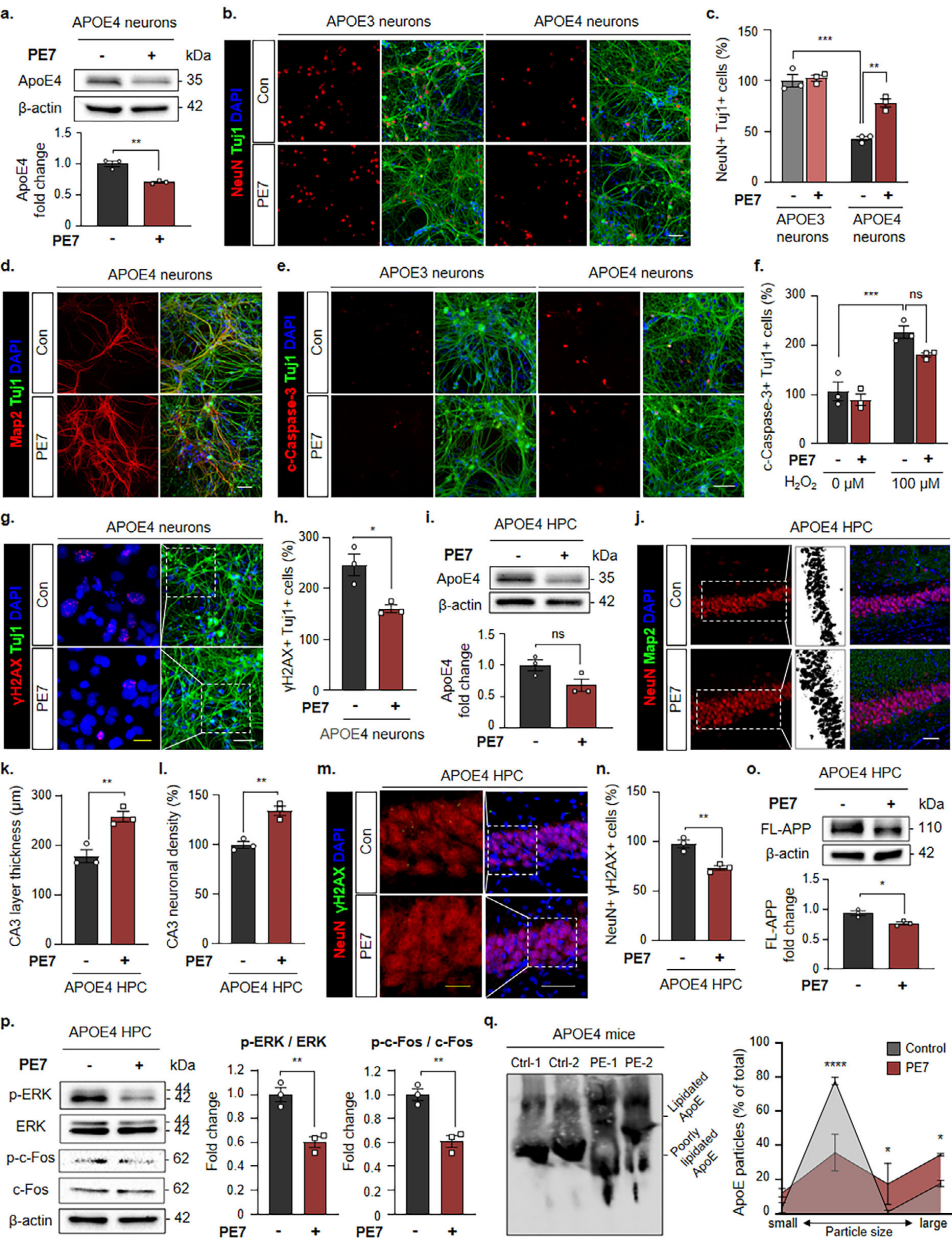
Mitigation of ApoE4‐associated neurotoxic phenotypes by prime editing. (a) Western blot analysis of human ApoE4 in primary neurons from APOE4 knock‐in (KI) mice with quantification, comparing control and PE‐treated groups. (b) Immunocytochemistry for NeuN (red), Tuj1 (green), and DAPI (blue) in primary neurons from APOE3 or APOE4 KI mice. Scale bar, 50 µm. (c) Quantification of NeuN‐ and Tuj1‐positive cells shown in (b). (d) Immunocytochemistry for Map2 (red), Tuj1 (green), and DAPI (blue) in APOE4 KI primary neurons. Scale bar, 50 µm. (e) Immunocytochemistry for cleaved caspase‐3 (c‐Caspase‐3, red), Tuj1 (green), and DAPI (blue) in APOE3 and APOE4 KI primary neurons. Scale bar, 50 µm. (f) Quantification of c‐Caspase‐3‐ and Tuj1‐positive cells shown in (e). (g) Immunocytochemistry for γH2AX (red), Tuj1 (green), and DAPI (blue) in APOE4 primary neurons. White scale bar, 50 µm; yellow, 20 µm. (h) Quantification of γH2AX‐ and Tuj1‐positive cells shown in (g). (i) Western blot analysis of ApoE4 in the hippocampus (HPC) of APOE4 KI mice with quantification. (j) Immunohistochemistry for NeuN (red), Map2 (green), and DAPI (blue) in the HPC of APOE4 KI mice. Scale bar, 50 µm. (k, l) Quantification of CA3 layer thickness (k) and neuronal density (l) based on (j). (m) Immunocytochemistry for NeuN (red), γH2AX (green), and DAPI (blue) in the HPC of APOE4 KI mice. White scale bar, 50 µm; yellow, 20 µm. (n) Quantification of γH2AX‐ and NeuN‐positive cells shown in (m). (o) Western blot analysis of full‐length APP (FL‐APP) in the HPC of APOE4 KI mice with quantification. (p) Western blot analysis of p‐ERK1/2 and p‐c‐Fos in the HPC of APOE4 KI mice with quantification. (q) Western blot analysis showing lipidated ApoE in PE‐treated APOE4 KI mice (left), with quantification of ApoE particle size (right). Data are presented as mean ± SEM. Statistical significance was determined by unpaired two‐tailed Student's t‐test (a, h, i, k, l, n, o, p), two‐way ANOVA with Tukey's (c, f), or Sidak's (q) multiple comparisons test. *p < 0.05, **p < 0.01, ***p < 0.001, ****p < 0.0001; ns, not significant. *n* = 3 biologically independent samples per group.

Next, we investigated PE7‐mediated editing of the APOE4 knock‐in (APOE4‐KI) allele in primary neurons from humanized APOE4‐KI mice, in which the endogenous mouse Apoe coding exons are replaced by the human APOE4 sequence. Following lentiviral delivery of PE7 into APOE4‐KI primary neuronal cultures, we observed a significant reduction in ApoE4 levels in the PE7‐treated group, indicative of efficient target editing in neuronal cultures (Figure 2a). To assess the functional efficacy of PE‐mediated editing, we initially examined whether PE7 treatment targeting APOE4 could reduce neuronal death. As expected, PE7 treatment significantly increased the number of NeuN‐ and Tuj1‐positive neurons in APOE4‐KI primary neuron cultures, with no observable effect in APOE3 controls (Figure 2b,c). In addition, enhanced neuronal survival was confirmed by elevated levels of Map2‐ and Tuj1‐positive cells in PE7‐treated APOE4 neurons (Figure 2d). We further observed that PE7‐treated neuronal cultures showed a trend toward attenuation of the H2O2‐induced increase in cleaved caspase‐3 (c‐Caspase‐3)‐positive and Tuj1‐positive cells, without reaching statistical significance (Figure 2e,f). In addition, PE7 treatment targeting APOE4 decreased γH2AX‐ and Tuj1‐double‐positive cells, indicating that PE‐mediated editing efficiently reduced oxidative stress‐induced apoptosis in APOE4‐KI neurons (Figure 2g,h). Taken together, these results indicate the efficient PE7‐mediated APOE4 correction in APOE4 neuronal cultures, resulting in a shift toward a lower‐risk APOE3 state and attenuation of ApoE4‐associated neurotoxic phenotypes.

To further evaluate the in vivo editing efficiency of PE7 targeting APOE4, the PE7 lentivirus was stereotaxically injected into the hippocampus of APOE4‐KI mice. Following stereotaxic delivery into the hippocampus of APOE4‐KI mice, PE7 expression was confirmed by immunoblotting for the Cas9 component of the editor (Figure S2b), and Sanger sequencing verified the intended C‐to‐T substitution at the APOE4 target locus (Figure S2d). Consistent with allele conversion rather than gene disruption, Western blot analysis of the hippocampal region showed a slight reduction in ApoE4 expression following PE7 treatment, supporting partial in vivo editing of the APOE4 allele (Figure 2i and Figure S2f). No detectable off‐target editing was observed at analyzed sites by deep sequencing (Figure S2a). Consistent with previous results, immunohistochemical analysis revealed an increased number of NeuN‐ and Map2‐positive neurons in the CA3 region of PE7‐treated APOE4‐KI mice, along with significant elevations in both CA3 layer thickness and neuronal density (Figure 2j‐l). In parallel, γH2AX and NeuN co‐immunostaining showed a marked decrease in γH2AX‐ and NeuN‐positive neurons, indicating that PE‐mediated APOE4 correction attenuated DNA damage in vivo (Figure 2m,n).

It has been reported that ApoE4 induces the transcriptional activity of the cAMP‐response element‐binding protein (CREB) by triggering the extracellular signal‐regulated kinase (ERK) cascade [16]. In turn, activated ERK1/2 can induce c‐Fos phosphorylation, which stimulates the production of APP and amyloid‐β [17]. Therefore, we examined whether PE7‐mediated editing of APOE4 could modulate APP expression in the hippocampus of APOE4‐KI mice. Western blot analysis revealed a significant reduction in APP protein levels following PE7 treatment (Figure 2o). Given that ApoE4 is known to selectively increase APP transcription, we next assessed the expression levels of these related genes. In line with this mechanism, PE7 treatment effectively suppressed App expression, while the expression levels of Aplp1 and Aplp2 remained unchanged (Figure S3a). Moreover, we reasoned that PE7‐mediated correction of the APOE4 allele could lead to APP suppression through attenuation of ERK1/2 signaling. Consistent with this idea, western blot analysis showed reduced levels of phosphorylated ERK1/2 (p‐ERK1/2) and phosphorylated c‐Fos (p‐c‐Fos) in hippocampal lysates from PE7‐treated APOE4‐KI mice (Figure 2p). Overall, these results suggest that PE7‐mediated genome editing of the APOE4 allele suppresses ApoE4‐dependent ERK1/2 signaling and may contribute to reduced Aβ production in ApoE4‐mediated AD. Furthermore, several studies underscore the importance of proper lipidation in the secretion of ApoE protein for its normal functions, including Aβ clearance [18]. In particular, ApoE4 exhibits reduced lipidation, which may result in insufficient lipid availability for function, especially in pathways involved in Aβ clearance. Thus, we examined the effects of in vivo PE treatment on lipidated ApoE protein levels using western blot analysis. Consistently, PE7 treatment reduced the proportion of small particles per total ApoE particles compared with the control, while the proportion of large particles increased, indicating enhanced lipidation of ApoE protein (Figure 2q). Collectively, these findings suggest that in vivo treatment with PE7 efficiently converted the APOE4 allele to the APOE3 allele, which can lead to alleviation of AD‐related molecular phenotypes observed in APOE4 mice.

### 2.3 Alleviation of AD‐Related Phenotypes by PE7‐Mediated Conversion of APOE4 to APOE3

**FIGURE 3:**
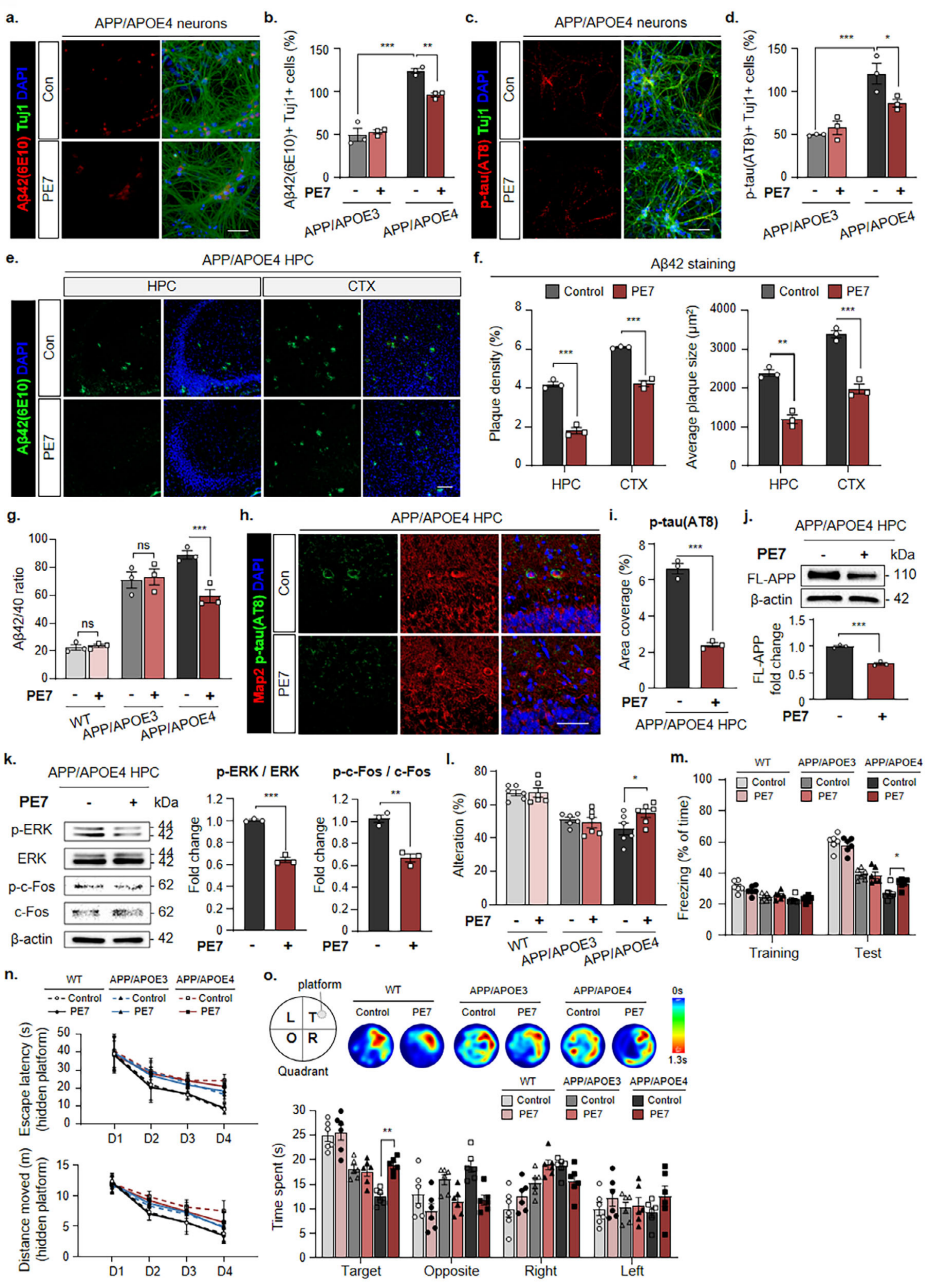
APOE4‐to‐APOE3 conversion–mediated attenuation of AD‐related phenotypes. (a) Immunocytochemistry for Aβ42 (6E10, red), Tuj1 (green), and DAPI (blue) in primary neurons from APP/APOE4 knock‐in (KI) mice, comparing control and PE‐treated groups. Scale bar, 50 µm. (b) Quantification of Aβ42 (6E10)‐ and Tuj1‐positive cells in APP/APOE3 and APP/APOE4 KI neurons under control or PE treatment. (c) Immunocytochemistry for p‐tau (AT8, red), Tuj1 (green), and DAPI (blue) in APP/APOE4 KI neurons, comparing control and PE‐treated groups. Scale bar, 50 µm. (d) Quantification of p‐tau (AT8)‐ and Tuj1‐positive cells in APP/APOE3 and APP/APOE4 KI primary neurons. (e) Immunohistochemistry for Aβ42 (6E10, green) and DAPI (blue) in the hippocampus (HPC) and cortex (CTX) of APP/APOE4 KI mice. Scale bar, 50 µm. (f) Quantification of Aβ plaque density (left panel) and average plaque size (right panel) in the HPC and CTX of APP/APOE4 KI mice shown in (e). (g) ELISA Quantification of hippocampal Aβ42/40 ratio in PE‐treated WT, APP/APOE3 KI and APP/APOE4 KI mice. (h) Immunohistochemistry for Map2 (red), p‐tau (AT8, green), and DAPI (blue) in the HPC of APP/APOE4 KI mice. Scale bar, 50 µm. (i) Quantification of p‐tau (AT8)‐positive area coverage (%) in the HPC of APP/APOE4 KI mice. (j, k) Western blot analysis of full‐length APP (FL‐APP) (j), p‐ERK1/2, and p‐c‐Fos (k) in the HPC of APP/APOE4 KI mice, comparing control and PE‐treated groups, with corresponding quantification. (l‐m) Behavioral outcomes in Y‐maze (l) and fear conditioning tests (m) in WT, APP/APOE3 and APP/APOE4 mice under control or PE treatment. (n) The escape latency (upper panel) and distance (lower panel) traveled during the Morris water maze trials in PE‐treated WT, APP/APOE3, APP/APOE4 mice. (o) Long‐term spatial reference memory assessed by quadrant occupancy heat maps (upper panel) and quantifications (lower panel) in the Morris water maze. Red indicates the most frequently visited areas. L, O, R, and T denote the left, opposite, right, and target quadrants, respectively; “Target” indicates the quadrant that previously contained the hidden platform, and the circle denotes its former location. Data are presented as mean ± SEM. Statistical significance was determined by unpaired two‐tailed Student's t‐test (f, i‐k), or two‐way ANOVA with Tukey's (b, d, m‐o) or Sidak's (g, l) multiple comparisons test. *p < 0.05, **p < 0.01, ***p < 0.001, ****p < 0.0001; ns, not significant. *n* = 3 per group, except (l–o) (*n* = 6; behavioral tests).

In the presence of mutant APP expression, ApoE4 has been implicated in exacerbating amyloid pathology by enhancing Aβ production and aggregation while simultaneously impairing its clearance and degradation, compared with ApoE3 or ApoE2 [19, 20]. Thus, we next investigated whether correction of APOE4 via PE7 could mitigate amyloid‐related phenotypes in primary neuronal cultures derived from APOE4‐KI mice harboring mutant APP (APP/APOE4‐KI). Consistently, we found that PE7 treatment significantly reduced the number of Aβ42‐ and Tuj1‐positive neurons in APP/APOE4‐KI primary cultures (Figure 3a,b). We further examined the accumulation of phosphorylated tau (p‐tau). Notably, PE7 treatment also led to a marked decrease in the number of p‐tau‐ and Tuj1‐positive neurons (Figure 3c,d), indicating that PE7 treatment effectively ameliorates amyloid‐related pathology and may also exert beneficial effects on tau‐related pathology.

We next examined whether in vivo conversion of APOE4 to APOE3 via PE7 could ameliorate amyloid‐related pathology in APOE4‐KI mice co‐expressing mutant APP (APP/APOE4‐KI mice), where the presence of ApoE4 in combination with mutant APP results in synergistically elevated Aβ levels and cognitive deficits compared with APP‐KI mice alone. PE7 expression in the hippocampus of APP/APOE4‐KI mice was similarly confirmed by immunoblotting for the Cas9 component of the editor following stereotaxic delivery (Figure S2c), and Sanger sequencing confirmed the intended C‐to‐T substitution at the APOE4 target locus following PE7 treatment (Figure S2e). ApoE4‐specific protein levels were also reduced while pan‐ApoE protein levels were maintained in APP/APOE4‐KI mice following PE7 treatment (Figure S2g). In PE7‐treated APP/APOE4‐KI mice, we observed a significant reduction in both the number and size of Aβ42 plaques in the hippocampus and cortex (Figure 3e,f). Furthermore, the Aβ42/Aβ40 ratio was decreased in PE7‐treated APP/APOE4‐KI mice (Figure 3g). In addition, immunohistochemical analysis revealed a reduction in p‐tau‐ and Map2‐double‐positive neurons, with a significant decrease in p‐tau‐stained area coverage following PE7 treatment (Figure 3h,i). Consistently, APP expression was also decreased in the hippocampi of PE7‐treated APP/APOE4‐KI mice (Figure 3j). Moreover, western blot analysis revealed a significant reduction in p‐ERK1/2 and p‐c‐Fos protein levels in PE7‐treated APP/APOE4‐KI mice (Figure 3k). Taken together, these results demonstrate that PE7‐mediated correction of APOE4 attenuates amyloid‐ and tau‐associated pathologies and modulates key disease‐related signaling pathways in vivo, thereby ameliorating AD‐related phenotypes.

Given that PE7 treatment alleviated disease‐associated molecular changes, including amyloid pathology, we next sought to determine whether these improvements could extend to cognitive function in the PE‐treated AD mouse model by conducting behavioral assays. First, a Y‐maze test was performed to assess the spatial and working memory of PE7‐treated APOE4‐KI mice with APP expression. The percentage of alternation decreased in the AD mouse group compared with WT mice, whereas lentiviral PE7‐injected APP/APOE4‐KI mice exhibited a significant increase in alternation compared with the control group (Figure 3l). Next, PE‐treated AD mice were subjected to the contextual fear conditioning test to assess short‐term memory. After 24 h of training, the freezing behavior of PE7‐treated APP/APOE4‐KI mice slightly increased (Figure 3m). In addition, we conducted the Morris water maze test to investigate long‐term spatial memory. During hidden platform training, the escape latency and distance traveled differed between WT mice and AD mice; however, both parameters in PE‐treated APP/APOE4‐KI mice were improved toward the levels observed in APP/APOE3‐KI mice (Figure 3n). In the probe test, the time spent in the target quadrant by PE7‐treated APP/APOE4‐KI mice significantly increased compared with control APP/APOE4‐KI mice (Figure 3o). These findings suggest that APOE4 correction alleviates APOE4‐associated pathological effects and partially restores hippocampus‐dependent memory performance in APP/APOE4‐KI mice. In line with this, localized hippocampal gene editing or lentiviral delivery has previously been shown to improve cognitive performance in AD mouse models [10, 21, 22], supporting the biological plausibility of the behavioral rescue observed in this study. Taken together, these results indicate that PE7 converting APOE4 to APOE3 restored the reduction of learning ability and attenuated memory impairment in APP/APOE4‐KI mice, suggesting that precise genome editing via prime editing may be a novel therapeutic strategy to rescue cognitive decline in AD mice.

### 2.4 PE7‐Mediated APOE4 Correction in AD Patient‐Derived Induced Neurons

**FIGURE 4:**
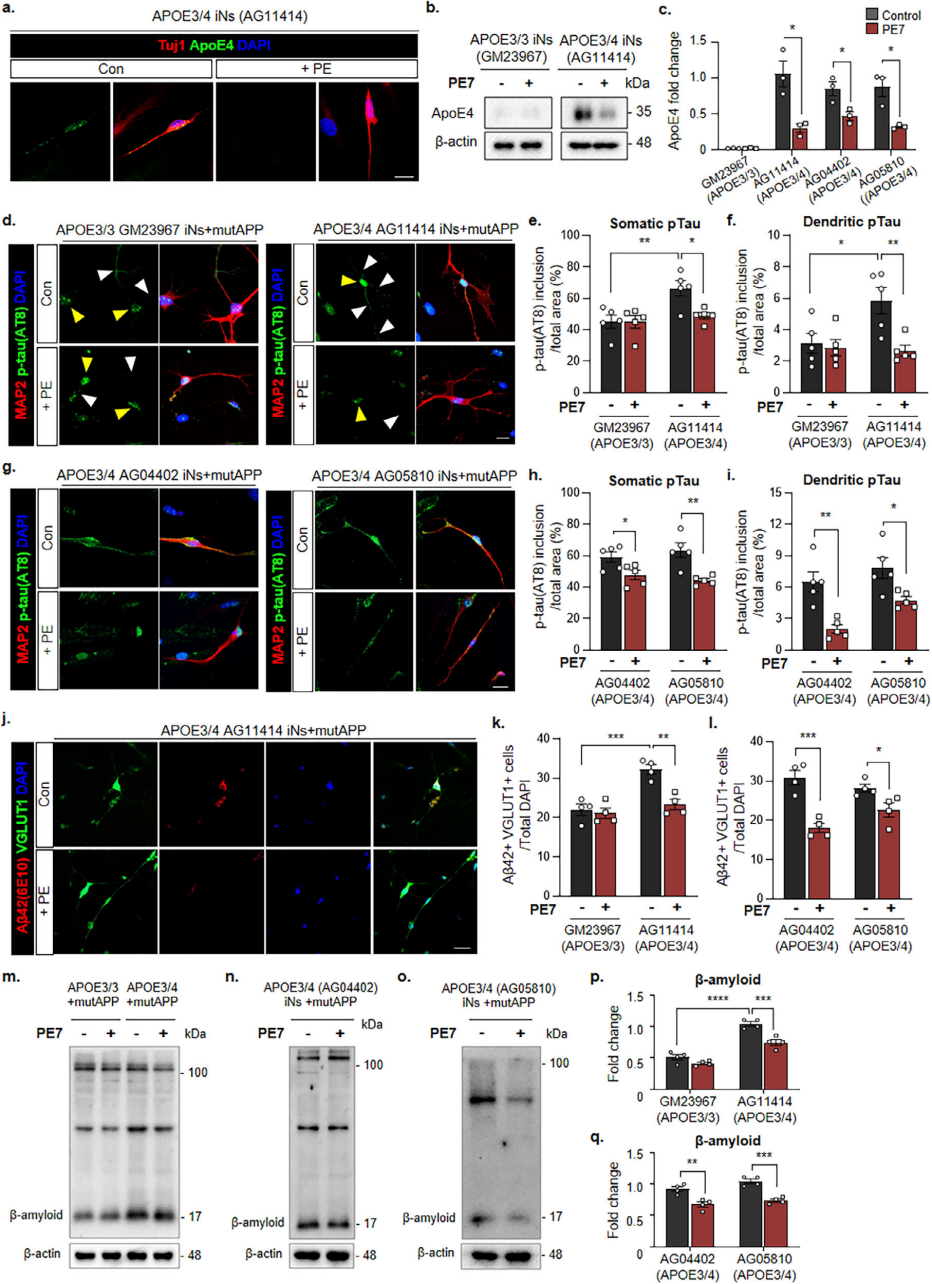
Functional validation of PE7‐mediated APOE4 correction in AD patient‐derived induced neurons. (a) Immunocytochemistry of induced neurons (iNs) directly reprogrammed from AD patient‐derived AG11414 fibroblasts, stained for Tuj1 (red), ApoE4 (green), and DAPI (blue), comparing control and PE‐treated groups. Scale bar, 20 µm. (b) Western blot analysis of human ApoE4 in iNs derived from AG11414 (APOE3/4, AD) and GM23967 (APOE3/3, healthy) fibroblasts under control or PE treatment. (c) Quantification of ApoE4 levels from iNs derived from AD patients (AG11414, AG04402, AG05810) and a healthy donor (GM23967). (d) Immunocytochemistry of mutAPP‐transduced iNs from GM23967 (APOE3/3, healthy) and AG11414 (APOE3/4, AD) fibroblasts, stained for MAP2 (red), p‐tau (AT8, green), and DAPI (blue). Somatic and dendritic p‐tau (AT8) inclusions are indicated by yellow and white arrows, respectively. Scale bar, 20 µm. (e, f) Quantification of somatic (e) and dendritic (f) p‐tau (AT8) inclusion as a percentage of total area in iNs from GM23967 (APOE3/3, healthy) and AG11414 (APOE3/4, AD), with or without PE treatment. (g) Immunocytochemistry of mutAPP‐transduced iNs from AG04402 and AG05810 (both APOE3/4, AD) fibroblasts, stained for MAP2 (red), p‐tau (AT8, green) and DAPI (blue). Scale bar, 20 µm. (h, i) Quantification of somatic (h) and dendritic (i) p‐tau (AT8) inclusion in iNs from AG04402 and AG05810 (both APOE3/4, AD) fibroblasts, with or without PE treatment. (j) Immunocytochemistry of iNs from AD patient‐derived AG11414 fibroblasts, stained for Aβ42 (6E10, red), VGLUT1 (green), and DAPI (blue). Scale bar, 20 µm. (k, l) Quantification of Aβ42‐ and VGLUT1‐positive cells in iNs from AD patient (AG11414, AG04402, AG05810) and a healthy donor (GM23967). (m) Western blot analysis of β‐amyloid in mutAPP‐transduced iNs from fibroblasts of a healthy donor (GM23967) and an AD patient (AG11414). (n, o) Western blot analysis of β‐amyloid in mutAPP‐transduced iNs from fibroblasts of AD patients (AG04402 and AG05810). (p, q) Quantification of β‐amyloid levels from western blot analyses shown in (m‐o), comparing control and PE‐treated groups. Data are presented as mean ± SEM. Statistical significance was determined by unpaired two‐tailed Student's t‐test (c, h, i, l, q) or two‐way ANOVA followed by Tukey's multiple comparisons test (e, f, k, p). *p < 0.05, **p < 0.01, ***p < 0.001, ****p < 0.0001; ns, not significant. *n* = 5 biologically independent samples per group for ICC data (e–f, h‐i); and *n* = 4 per group for ICC data (k‐l) and WB data (p‐q); except (c) (*n* = 3).

While mouse models carrying knock‐in human APOE4, including APP/APOE4 disease models, provide valuable insights into the molecular and pathological consequences of ApoE4, they remain genetically engineered systems that do not fully recapitulate the genetic background and epigenetic complexity of human patients. To assess the translatability of our findings, we next examined the efficacy of PE7‐mediated APOE4 correction in induced neurons (iNs) directly reprogrammed from APOE3/4 AD patient‐derived fibroblasts (Table S2). Specifically, fibroblasts from AD patients were transduced with a lentivirus constitutively expressing Ascl1, Brn2, and Myt1l, together with the PE7 lentivirus targeting APOE4. In agreement with previous data, immunocytochemistry showed that PE7 treatment led to a marked reduction in ApoE4 signal in iNs derived from APOE3/4 AD patient fibroblasts, indicating efficient editing at the protein level (Figure 4a and Figure S4a). Moreover, western blot analysis showed that ApoE4 protein was markedly reduced in APOE3/4 AD patient‐derived iNs following PE7 treatment, while APOE3/3‐derived iNs showed no detectable signal (Figure 4b and Figure S4b). To confirm the consistency of this effect across multiple genetic backgrounds, we evaluated ApoE4 protein levels in iNs from additional APOE3/4 patient fibroblasts. Similarly, additional AD patient iNs with the APOE3/4 allele exhibited significant reductions in ApoE4 protein levels following PE7 treatment (Figure 4c).

We next examined phosphorylated tau (p‐tau) accumulation in these patient iNs that express the APP mutant. Compared with APOE3/3‐derived iNs, APOE3/4 AD patient‐derived iNs showed stronger p‐tau signals under vehicle‐treated conditions, which were markedly reduced following PE7 treatment (Figure 4d,g). Hyperphosphorylated tau is known to accumulate in both somatic and dendritic compartments, representing a defining feature of tau mislocalization in AD and related tauopathies [23]. Given the compartment‐specific distribution of p‐tau in AD, we separately analyzed its somatic and dendritic accumulation. Consistent with the qualitative findings, APOE3/3 iNs showed minimal p‐tau levels with no significant change upon PE7 treatment, whereas PE7‐treated APOE3/4 iNs displayed significant reductions in both somatic and dendritic p‐tau intensity (Figure 4e,f,h,i). These results suggest that PE7 treatment led to a clear reduction in p‐tau inclusion, with significant decreases in both somatic and dendritic compartments.

Next, to assess whether PE7‐mediated APOE4 correction also influences amyloid‐related synaptic pathology, we examined Aβ42 accumulation in these APP mutant‐expressing iNs. Immunocytochemistry using Aβ42 and VGLUT1 was performed in iNs derived from APOE3/3 and APOE3/4 fibroblasts. While PE7 treatment had no observable effect in APOE3/3 iNs, APOE3/4 iNs showed an increased number of Aβ42^+^/VGLUT1^+^ double‐positive neurons under control conditions, which were significantly reduced following PE7 treatment (Figure 4j,k). This pattern was also consistently observed in two additional APOE3/4 patient‐derived iN lines, confirming the reproducibility of the effect across different genetic backgrounds (Figure 4l). To further evaluate Aβ‐related changes at the protein level, we analyzed β‐amyloid expression by western blot in iNs derived from APOE3/3 and three APOE3/4 patient fibroblast lines (Figure 4m–o). β‐amyloid was considerably lower in APOE3/3 iNs, and PE7 treatment did not alter its level (Figure 4m,p). In contrast, APOE3/4‐derived iNs showed elevated β‐amyloid levels under control conditions, which were significantly reduced by PE7 treatment (Figure 4n,o,q). Taken together, these results demonstrate that PE7‐mediated APOE4 correction effectively reduces ApoE4 protein levels and alleviates tau‐ and amyloid‐related AD pathologies in iNs derived from multiple APOE3/4 AD patient lines, supporting the therapeutic potential and translational relevance of this strategy.

## 3 Discussion

Alzheimer's disease (AD) is a progressive neurodegenerative disorder and the leading cause of dementia worldwide, marked by cognitive decline and pathological hallmarks such as amyloid‐β (Aβ) plaques and tau tangles [1]. While rare familial forms are linked to mutations in APP or presenilins, most AD cases are sporadic, with the APOE4 allele representing the strongest genetic risk factor [24]. Given the critical role of ApoE4 in sporadic Alzheimer's disease and its association with increased amyloid burden, neuroinflammation, and earlier disease onset, developing precise allele‐specific therapeutic strategies has remained a major objective in AD research. Here, we developed an APOE4‐specific prime editing strategy, based on an optimized APOE4‐targeting pegRNA combined with the PE7 system, to convert the high‐risk APOE4 allele into the lower‐risk APOE3 variant as a therapeutic strategy for ApoE4‐mediated sporadic AD. We showed that lentiviral delivery of PE7 enabled precise and allele‐specific editing of the APOE4 allele, resulting in its conversion to APOE3 across multiple experimental models, including murine cell lines, primary neurons, and human induced neurons reprogrammed from APOE3/4 AD patient fibroblasts. This correction led to a reduction in ApoE4 protein levels and alleviated hallmark AD‐related phenotypes, including Aβ42 accumulation and tau phosphorylation. These molecular improvements were further accompanied by enhanced neuronal survival and cognitive function in an AD mouse model harboring both APOE4 and mutant APP alleles. Furthermore, the therapeutic effect was reproducibly observed in human induced neurons derived from diverse APOE3/4 patient lines, supporting the translational relevance of this approach. Taken together, these findings demonstrate that prime editing can serve as a high‐precision genome editing platform for mitigating ApoE4‐driven AD pathology and advancing allele‐specific therapeutic strategies for sporadic AD. Beyond its therapeutic relevance in Alzheimer's disease, our findings further support the broader applicability of prime editing as a versatile therapeutic genome‐engineering platform. Previous studies have demonstrated successful prime editing–mediated correction of pathogenic variants in diverse disease settings, including HBB‐associated sickle cell disease, CFTR‐linked cystic fibrosis, Atp1a3‐associated alternating hemiplegia of childhood, and Pde6b‐driven retinitis pigmentosa [25, 26, 27, 28]. These reports collectively established the feasibility of therapeutic prime editing across hematologic, epithelial, neurological, and retinal systems. In this broader context, our results extend the therapeutic scope of prime editing into a sporadic neurodegenerative disease driven by a common risk allele, demonstrating that moderate yet precise APOE4‐to‐APOE3 correction is sufficient to confer meaningful molecular and functional rescue.

Previous studies have demonstrated the feasibility of converting the APOE4 allele to APOE3 using prime editing [29, 30]. In particular, Günaydin et al. recently demonstrated CNS‐directed in vivo prime editing of human APOE4 to APOE3 using split‐intein AAV vectors, providing important proof‐of‐concept for brain‐wide allele correction. However, no study to date has assessed whether such correction can mitigate molecular and functional hallmarks of AD in vivo. Thus, our findings advance this field by providing the first evidence that PE7‐mediated APOE4 correction not only reduces ApoE4 protein expression but also alleviates key pathological features—including Aβ accumulation, tau phosphorylation, and ERK1/2 signaling—in primary neurons, AD model mice, and patient‐derived iNs. Furthermore, the ability of PE7 to rescue cognitive impairment in a humanized AD mouse model highlights the functional relevance and therapeutic promise of allele‐specific editing strategies targeting APOE4.

In addition to its biological impact, our study highlights several advantages of the PE7 system for therapeutic genome editing. The incorporation of the La protein's RNA‐binding domain into PE7 improves pegRNA stability, thereby enhancing editing efficiency, particularly in systems where pegRNA expression may be limiting. The observed editing precision—without detectable off‐target mutations—further supports the safety of this approach. Importantly, we found that moderate levels of gene editing in APOE4 were sufficient to confer phenotypic improvements in ApoE4‐mediated Alzheimer's disease. Consistent with previous studies demonstrating that prime editing can exert functional benefits at moderate editing efficiencies [27, 28], our results underscore its potential for therapeutic application in a wide range of genetic disorders. Despite these promising outcomes, several technical challenges remain to be addressed before clinical application. While lentiviral vectors enabled proof‐of‐concept editing in this study, clinically viable delivery systems—such as AAV or adenoviral vectors—are constrained by limited cargo capacity. Recent developments involving dual‐AAV delivery of split‐intein prime editors may offer a potential workaround, though further optimization is required. Enhancing delivery specificity, minimizing immunogenicity, and improving editing kinetics will be critical for translating prime editing into a therapeutic platform for neurological diseases.

In summary, our findings provide compelling preclinical evidence that prime editing can be harnessed for allele‐specific correction of APOE4, thereby ameliorating both molecular and functional phenotypes in AD models. The successful application of PE7 in both mouse and human‐derived systems underscores its potential as a targeted genome editing strategy for sporadic AD. Future studies should aim to optimize delivery systems and assess long‐term safety and efficacy in more complex models to pave the way for clinical translation.

## 4 Methods

### 4.1 Preparation of Plasmids

The PE7‐encoding plasmid was constructed by fusing the N‐terminal domain of La (1‐194) from pT7‐PE7 (Addgene #214813) into the pLenti‐PE2max‐BSD (Addgene #191102) backbone via Gibson assembly. Lentiviral plasmids encoding both PE and the 121‐bp APOE4 target sequence were generated by inserting the target sequence, amplified from mouse fibroblast genomic DNA, into the PE lentiviral plasmid using Gibson assembly. epegRNA‐encoding sequences were cloned into either Lenti_gRNA‐Puro vector (Addgene #84752) or pU6‐tmpknot‐GG‐acceptor vector (Addgene #174039) via Golden Gate assembly. A schematic representation of the lentiviral constructs used in this study is provided in Figure S1b.

### 4.2 Cell Culture

HEK293T (ATCC), NIH3T3 (ATCC) and mouse fibroblasts were cultured in Dulbecco Modified Eagle Medium (DMEM, Gibco) supplemented with 10% fetal bovine serum (FBS, RDT) and 1X Penicillin‐Streptomycin (P/S, Thermo Fisher Scientific). Primary neurons were derived from APOE3, APOE4, APP/APOE3 and APP/APOE4 mice on embryonic day 14 (E14) and cultured in neurobasal medium (Gibco, Waltham, MA), supplemented with heat‐inactivated fetal bovine serum (Gibco), glutamine (Gibco), B‐27 supplement (Gibco), L‐glutamine (Gibco), P/S (Gibco), and laminin (Corning, Corning, NY). Primary neurons were maintained for 2 weeks before undergoing biochemical analysis. All cells were incubated at 37°C in 5% CO_2_. Authentication of cell lines was conducted through STR (Short Tandem Repeat) analysis (Kogene Biotech, Seoul, Korea), and mycoplasma testing was performed using a MycoSensor PCR assay kit (Agilent, Santa Clara, CA).

### 4.3 Lentivirus Production

To produce lentivirus encoding each of the 40 individual pegRNAs, 1.0 × 10^6^ HEK293T cells were seeded per well in 6‐well culture plates (SPL Life Sciences). To produce lentiviruses encoding PE or PE‐APOE4 target sequence, 12.0 × 10^6^ HEK293T cells were seeded per 150‐mm dish (SPL Life Sciences). After 20 h, the medium was replaced with fresh medium, and a plasmid mixture containing transfer plasmid, psPAX2 (Addgene #12260), and pMD2.G (Addgene #12259) at a weight ratio of 4:3:1 (total DNA of 3.2 µg for 6‐well plates or 40 µg for 150‐mm dishes) was transfected into HEK293T cells using PEI MAX (Polysciences). The medium was replaced with fresh medium 20 h post‐transfection. Lentivirus‐containing supernatants were harvested 48 h after transfection. For lentivirus collected from 6‐well plates, supernatants were centrifuged at 4750 rpm for 30 min at 4°C, and the final supernatant was collected and stored at −80°C. For lentivirus harvested from 150‐mm dishes, supernatants were filtered through 0.45‐µm pore size filters (Millipore) before storage at −80°C. For in vivo stereotaxic injections, lentiviral supernatants were further concentrated by ultracentrifugation at 25 000 rpm for 2 h at 4°C. After complete removal of the supernatant, viral pellets were resuspended in cold PBS and used for subsequent experiments. Functional titers were determined by transducing HEK293FT cells with serial dilutions of virus, followed by quantification of GFP‐positive cells 48–72 h post‐transduction using flow cytometry. To ensure accurate titer estimation, only dilution factors yielding 1–20% GFP positivity were included in the analysis. Titers were calculated using the following formula: titer (TU mL^−1^) = (initial cell number × % GFP‐positive cells × dilution factor) / inoculum volume. The final concentrated lentiviral preparations used for in vivo injections had a functional titer of approximately 1 × 10^8^ TU mL^−1^.

### 4.4 Evaluation of Prime Editing System Activities in Mouse Fibroblasts and NIH3T3 Cells

To establish PE‐expressing fibroblasts, mouse fibroblasts were transduced with lentivirus encoding PE at a multiplicity of infection (MOI) of 5 in the presence of 8 µg mL^−1^ of polybrene (Sigma–Aldrich). After 20 h, the medium was replaced with DMEM containing blasticidin (20 µg mL^−1^) for selection, followed by maintenance in DMEM containing blasticidin (10 µg mL^−1^). For pegRNA activity validation, PE‐expressing mouse fibroblasts were seeded at 5 × 10^4^ cells per well in 48‐well plates and transduced with epegRNA‐encoding lentivirus at an MOI of 0.3 with 8 µg mL^−1^ polybrene. The medium was replaced with DMEM containing blasticidin (10 µg mL^−1^) and puromycin (2 µg mL^−1^), and cells were harvested 7 days post‐transduction. NIH3T3 cells were transduced with lentivirus encoding both PE and APOE4 target sequence at a MOI of 0.3 in the presence of 8 µg mL^−1^ of polybrene. Selection and maintenance was performed as described for mouse fibroblasts. For transduction‐based evaluation, established NIH3T3 cell lines were seeded at 1.5 × 10^5^ cells per well in 24‐well plates and transduced with epegRNA‐encoding lentivirus at a MOI of 0.3 after 20 h. The medium was replaced with DMEM containing blasticidin (10 µg mL^−1^) and puromycin (2 µg mL^−1^), and cells were harvested 7 days post‐transduction. For plasmid transfection‐based evaluation, established NIH3T3 cell lines were seeded at 1.0 × 10^5^ cells per well in 6‐well plates and transfected with pegRNA‐encoding plasmids (1 µg) using Lipofectamine 2000 (Thermo Fisher) according to the manufacturer's instruction. To facilitate selection, pEGIP plasmid (Addgene #26777, 200 ng) was co‐transfected with pU6‐tmpknot‐GG‐acceptor‐based pegRNA plasmids. The medium was replaced with DMEM containing blasticidin (10 µg mL^−1^) and puromycin (2 µg mL^−1^), and cells were harvested 5 days post‐transfection.

### 4.5 Targeted Deep Sequencing Analysis

Target genomic regions were amplified using a two‐step PCR protocol. Primer sequences are listed in Table S3. For PE‐integrated mouse fibroblasts, cells were lysed in 100 µL of lysis buffer (10 mM Tris‐HCl, pH 7.0, 0.05% SDS, and 25 µg mL^−1^ proteinase K) at 37°C for 1 h, followed by enzyme inactivation at 80°C for 15 min. The first PCR was performed in a 50 µL reaction containing 25 µL of 2X Taq PCR Smart Mix (SolGent), 5 µL of the cell lysate, and 10 pmol of each primer. Cycling conditions were as follows: 95°C for 1 min, 35 cycles of 95°C for 1 min, 60°C for 30 s, and 72°C for 30 s, followed by a final extension at 72°C for 7 min. To attach Illumina adaptor sequences, a second PCR was conducted at a total volume of 50 µL with 25 µL of 2X Taq PCR Smart Mix, 1 µL of the first PCR product, and 10 pmol of each primer using the following conditions: 95°C for 1 min, followed by 15 cycles of 95°C for 30 s, 60°C for 30 s, 72°C for 30 s, followed by a final extension 72°C for 7 min. For other samples, genomic DNA was extracted using the AccuPrep Genomic DNA Extraction Kit (BIONEER) according to the manufacturer's protocol. The first PCR was conducted in a 30 µL reaction containing 15 µL of 2X Taq PCR Smart Mix, at least 30 ng of genomic DNA, and 10 pmol of each primer. Cycling conditions were identical to those described above, except for the number of cycles depending on the target region: 25 cycles for on‐target sites, 30 cycles for potential off‐target sites except for sites 15 and 16, and 35 cycles for off‐target sites 15 and 16. The second PCR was performed in a 30 µL reaction containing 15 µL of 2X Taq PCR Smart Mix, 1 µL of the first PCR product, and 10 pmol of each primer, using the same cycling conditions as above. The final amplicons were gel‐purified using MEGAquick‐spin Total Fragment DNA Purification kit (iNtRON Biotechnology). Targeted deep sequencing was conducted using the iSeq 100 Sequencing System (Illumina) and the data was analyzed by CRISPR RGEN tools [31] (http://www.rgenome.net/).

### 4.6 Animal Experiments

All animal experiments were approved by the Institutional Animal Care and Use Committee of Dongguk University and performed in accordance with institutional guidelines (IACUC‐2022‐049‐3). APOE3 KI (B6.Cg‐Apoe^em2(APOE*)Adiuj^/J; # 029018) and APOE4 KI (B6(SJL)‐Apoe^1.1(APOE*4)Adiuj^/J; #027894) transgenic mice were acquired from The Jackson Laboratory. These strains are humanized APOE knock‐in models in which the endogenous mouse Apoe coding exons are replaced by the corresponding human APOE3 or APOE4 sequence. APP KI (AppNL‐G‐F/NL‐G‐F; Swedish (NL), Arctic (G), and Beyreuther/Iberian (F) mutation; #RBRC06344) transgenic mice were obtained from RIKEN Brain Science Institute [32]. APP/APOE3 and APP/APOE4 mice were generated by crossing APP KI mice with APOE3 KI or APOE4 KI mice. Accordingly, all compound models retain the same humanized APOE knock‐in background. For stereotaxic injection, mice were anesthetized with 120 mg/kg of Avertin (2,2,2‐tribromoethanol; Sigma, St. Louis, MO). A total volume of 5 µL per hemisphere was microinjected into the hippocampus of each hemisphere at the following coordinates: AP −2.06 mm, ML ±2.5 mm, and DV −2.5 mm. The control group received 2.5 µL of pLenti‐PE7‐BSD only supplemented with 2.5 µL of sterile saline, whereas the PE7 + pegRNA group received 2.5 µL each of pLenti‐PE7‐BSD and pegRNA lentivirus. The final lentiviral preparation was adjusted to 1 × 10^8^ TU mL^−1^. Injections were performed twice at a one‐week interval using the same stereotaxic coordinates, injection volume, and viral dose. After injection, mice were kept warm and monitored until fully awake. Behavioral tests and biochemical analyses were conducted one month after the first injection [10, 33]. Behavioral assays were conducted on 7‐month‐old APOE4‐KI mice and APOE3‐KI mice (n = 6 males per group), 4‐month‐old APP/APOE4 KI‐mice and APP/APOE3 KI‐mice (n = 6 males per group), including the Y‐maze, fear conditioning, and Morris water maze assays. The Y‐maze assay tested alternation in a Y‐shaped three‐open arm maze, recording mouse behaviors for 10 mins and calculating the total number of spontaneous visits to each arm position. Fear conditioning tests were conducted over two consecutive days, with day 1 involving exploration in a fear conditioning chamber for 3 min, followed by exposure to paired stimuli and aversive unconditioned stimuli (2 s, 0.2 mA). On day 2, freezing behavior tests were conducted for 2 min to evaluate conditioned fear. Morris water maze tests were performed in a circular pool with a submerged platform, and mouse behavior was recorded during 60‐s trials. During the acquisition phase, mice were trained to locate the hidden platform over four consecutive days, with three trials per day. For the assessment of spatial memory, a probe test was conducted 24 h after the final training session by removing the platform and allowing mice to swim freely for 60 s. During the probe trial, the time spent in each quadrant was recorded and analyzed. Observations for all behavioral tests, including the Y‐maze, fear conditioning, and water maze tests, were recorded and analyzed using Noldus Ethovision XT 13 (Noldus, Netherlands). Mice were randomly chosen for behavioral tests. Following the behavioral assessments, each male mouse was sacrificed for biochemical analysis. Both the behavioral tests and biochemical analyses were conducted independently and in a blinded manner.

### 4.7 Ethics Statement

All experimental procedures and animal care in this study were carried out in accordance with the guidelines approved by the Institutional Animal Care and Use Committee of Dongguk University (IACUC‐2022‐049‐3).

### 4.8 Immunofluorescence Analysis

Following the previously established protocol [34], primary neurons and hippocampal brain tissues from AD mice were fixed in 4% paraformaldehyde (Sigma) and washed with PBS buffer twice. The samples were blocked with PBST supplemented with 1% BSA for 20 min and incubated with primary antibodies (anti‐Cleaved Caspase‐3 antibodies [Cell signaling, #9661S]; anti‐βIII‐tubulin antibodies [Sigma–Aldrich, T8578]; anti‐ NeuN antibodies [Invitrogen, PA5‐78639]; anti‐ Map2 antibodies [Thermo Fisher Scientific, 13‐1500]; anti‐beta Amyloid 1–42 antibodies [Abcam, ab201060]) at 4°C overnight. Then they were washed with PBST, incubated with appropriate secondary antibodies at RT for 2 h, and counterstained with DAPI (Invitrogen). The stained samples were visualized under an LSM 700 confocal microscope (Zeiss, Oberkochen, Germany).

### 4.9 Sanger Sequencing

On‐target sequence was amplified by PCR and cloned into a pTOP TA V2 vector through TOPcloner TA Core Kit (Enzynomics, Daejeon, Korea). Sequencing of TA‐cloned products was conducted using an M13 reverse primer (5′‐GCG GAT AAC AAT TTC ACA CAG‐3′).

### 4.10 RNA Isolation and Quantitative Real‐time PCR Analysis

RNA extraction from primary mouse neurons and brain tissues was performed using the eCube tissue RNA mini kit (Philekorea, Seoul, Korea) following the manufacturer's instructions. Briefly, 1 µg of the prepared RNA was reverse‐transcribed using AccuPower CycleScript RT PreMix (Bioneer, Daejeon, Korea). Subsequent qRT‐PCR was carried out on a Rotor‐Gene Q real‐time PCR cycler (Qiagen, Hilden, Germany) with appropriate primer sets and AccuPower PCR PreMix (Bioneer, Daejeon, Korea). Gene expression for each marker was normalized against GAPDH in each sample.

### 4.11 Beta‐Amyloid Quantification

Samples, including cells and brain tissues, were treated with RIPA buffer (Sigma–Aldrich) and 1× proteinase inhibitor cocktail (Sigma). The levels of intracelluar Aβ40 and Aβ42 were assessed utilizing Aβ (1–40) and Aβ (1–42) (FL) kits (IBL International, Hamburg, Germany), respectively, following the manufacturer's protocols. Absorbance at 450 nm was quantified using a VERSAmax tunable microplate reader (Molecular Devices, San Jose, CA).

### 4.12 Western Blot Analysis

For western blot analysis, samples were lysed with RIPA buffer (1% NP‐40, 0.5% DOC, 0.1% SDS, and 150 mmol/L NaCl in 50 mmol/L Tris, pH 8.0; Sigma–Aldrich, R0278) and 1x proteinase inhibitor, followed by homogenization. Subsequently, the samples were mixed with 5x loading buffer and boiled at 100°C for 10 mins. 10 µg of extracted proteins were separated via 12% sodium dodecyl sulfate‐polyacrylamide gel electrophoresis (SDS‐PAGE) and transferred onto a nitrocellulose membrane (GE Healthcare, 10600001). Transferred membrane were incubated with appropriate primary anti‐Phospho‐p44/42 MAPK antibodies (Cell Signaling Technology, #4370), anti‐p44/42 MAPK antibodies (Cell signaling Technology, #4695), anti‐Phospho‐c‐Fos antibodies (Cell signaling Technology, #5348), anti‐c‐Fos antibodies (Cell signaling Technology, #2250), anti‐APP A4 antibodies (Sigma, MAB348), anti‐Apolipoprotein E4 antibodies (Sigma, MABN43), anti‐APP C‐terminal antibodies (Sigma, A8717), anti‐ApoE antibodies (Invitrogen, 701241) and and anti‐beta‐actin antibodies (Abfrontier, LF‐PA0207) overnight at 4°C. After incubation, samples were washed with PBS and then incubated with suitable HRP‐conjugated secondary antibodies for 2 h at room temperature. The resulting bands were visualized using an ECL kit (Dogen, DG‐WF200). Band quantification was performed using ImageJ (NIH) software. All experiments were conducted at least three times, and the experimenter was not blinded to the treatment. No experiments were excluded from the analyses.

### 4.13 Statistical Analysis

All data are presented as the mean ± SEM of three or more independent experiments; n values represent the number of individual experiments performed or mice; Dots indicate the number of independent experiments or mice. In each in vitro experiment, a minimum of three technical replicates of independent experiments were conducted to ensure sufficient statistical power. Statistical analyses were performed using SPSS version 18.0 (IBM Corporation). Group differences were considered statistically significant at *p < 0.05, **p < 0.01, and ***p < 0.001. Significant intergroup differences were assessed using a two‐way analysis of variance (ANOVA) followed by Tukey's or Sidak's multiple comparisons test for post‐hoc analysis, and two‐tailed Student's t‐tests for two‐component comparisons after confirmation of a normal distribution. The behavior and histological image analyses of PE‐treated mice were conducted in a blinded manner by independent investigators. No statistical methods were used to predetermine sample sizes; nevertheless, our sample sizes were similar to those documented in previous publications [10, 35, 36, 37]. Data collection and analysis procedures were randomized and blinded.

## Author Contributions

H.P., and J.P.K. conceptualized the study. Y.K., G.L., S.A., H.P., H.W.K., J.S.K., S.K., S.M.K., J.P., S.H.K. conducted the investigations. Y.K., G.L., H.P., H.W.K. and J.S.K. designed the methodology. Y.K., G.L., H.W.K., H.P., S.K. and Y.H. validated the results. X.Y., J.J. and H.L performed further validation. Y.K., S.A., G.L., H.W.K., H.P. and D.K. prepared the visualizations. Y.K., G.L., H.W.K., H.K.K., J.P.K. wrote the original manuscript. Y.K., G.L., J.S.K., S.A., H.K.K., J.P.K. reviewed and edited the manuscript. H.K.K. and J.P.K. supervised the project and oversaw its administration. J.P.K. secured financial support for the study. All authors contributed to the writing of this manuscript and have approved the final version.

## Conflicts of Interest

The authors declare no conflicts of interest.

## Supporting information




**Supporting File 1**: advs76658‐sup‐0001‐SuppMat.pdf.

**Supporting File 2**: advs76658‐sup‐0002‐SuppTable.pdf.

## Data Availability

The data that support the findings of this study are available from the corresponding author upon reasonable request.
