# Supplementary material for: Therapeutic Gene Editing of APOE4 in Sporadic Alzheimer's Disease via Prime Editor 7
Source: Adv Sci (Weinh). 2026 Jul 17;13(57):e76658. doi: 10.1002/advs.76658 (PMC13379251; doi:10.1002/advs.76658)
Supplement: Supplementary file 1 [file ADVS-13-e76658-s001.pdf]

Supplementary Table 1. Information of all pegRNAs used in this study

Guide index	Guide Sequence (G/N19)	RTT Sequence	PBS Sequence	linker	RTT length	PBS length	co-edit type
1	GGCGCAGGCCCGGCTGGGCG	GGCCGCaCACGTCCTCCATGTCCGCGC	CCAGCCGGGC	TTAATATC	27	10	-
1	GGCGCAGGCCCGGCTGGGCG	GGCCGCaCACGTCCTCCATGTCtGCGC	CCAGCCGGGC	ATATAATT	27	10	A
1	GGCGCAGGCCCGGCTGGGCG	GCGGCCGCaCACGTCCTCCATGTCtGCGC	CCAGCCGGGC	GAATAATT	29	10	A
1	GGCGCAGGCCCGGCTGGGCG	TGCACCAGGCGGCCGCaCACGTCCTCCATGTCtGCGC	CCAGCCGGGC	TTATATAT	37	10	A
1	GGCGCAGGCCCGGCTGGGCG	AGGCGGCCGCaCACGTCCTCCATGTCtGCGC	CCAGCCGGGC	TAAATTAT	31	10	A
2	GGCCCGGCTGGGCGCGGACA	GGCCGCaCACGTCCTCCATGT	CCGCGC	AGAATCAT	21	6	-
2	GGCCCGGCTGGGCGCGGACA	GCGGCCGCaCACGTCCTCCATGT	CCGCGC	GAAATAAA	23	6	-
2	GGCCCGGCTGGGCGCGGACA	GGCCGCaCACGTCCTCCATGT	CCGCGCC	ATATTATA	21	7	-
2	GGCCCGGCTGGGCGCGGACA	GGCGGCCGCaCACGTCCTCCATGT	CCGCGC	GAATACAC	24	6	-
2	GGCCCGGCTGGGCGCGGACA	GGCCGCaCACGTCCTCCATGT	CCGCGCCCAG	GAATTTAA	21	10	-
3	GCGGCTGGGCGCGGACATGG	GGCCGCaCACGTCCTCCA	TGTCCGCGC	GATAATTC	18	9	-
3	GCGGCTGGGCGCGGACATGG	GGCCGCaCACGTCtTCCA	TGTCCGCGC	AATTTACA	18	9	B
3	GCGGCTGGGCGCGGACATGG	GCGGCCGCaCACGTCtTCCA	TGTCCGCGC	ATTTAATC	20	9	B
3	GCGGCTGGGCGCGGACATGG	GGCCGCaCACGTCtTCCA	TGTCCGCGCC	ATATTAAT	18	10	B
3	GCGGCTGGGCGCGGACATGG	GGCGGCCGCaCACGTCtTCCA	TGTCCGCGC	AAATTATT	21	9	B
3	GCGGCTGGGCGCGGACATGG	GCGGCCGCaCACGTCtTCCA	TGTCCGCGCC	ATTTAAGA	20	10	B
4	GCGGACATGGAGGACGTGCG	AGGCGGCCGCaC	ACGTCCTCCATG	ATAAATTT	12	12	-
4	GCGGACATGGAGGACGTGCG	AGGCGGCCGCat	ACGTCCTCCATG	AAATAGAC	12	12	C
4	GCGGACATGGAGGACGTGCG	AGGCGGCCGCag	ACGTCCTCCATG	ATAAATAT	12	12	D
4	GCGGACATGGAGGACGTGCG	AGGCGGCCGCaa	ACGTCCTCCATG	AATAAACC	12	12	E
4	GCGGACATGGAGGACGTGCG	AGGCGGCCaCaC	ACGTCCTCCATG	AATATAAA	12	12	F
4	GCGGACATGGAGGACGTGCG	AGGCGGCCaCat	ACGTCCTCCATG	AATATAAC	12	12	G
4	GCGGACATGGAGGACGTGCG	AGGCGGCCaCag	ACGTCCTCCATG	ATAATAAA	12	12	H
4	GCGGACATGGAGGACGTGCG	AGGCGGCCaCaa	ACGTCCTCCATG	AAATTAAC	12	12	I
4	GCGGACATGGAGGACGTGCG	AGGCGtCCaCat	ACGTCCTCCATG	AATTATTA	12	12	J
4	GCGGACATGGAGGACGTGCG	GCGGCCGCaC	ACGTCCTCCATG	AAATACTA	10	12	-
4	GCGGACATGGAGGACGTGCG	GGCGGCCGCaC	ACGTCCTCCATG	ATAAATAT	11	12	-
4	GCGGACATGGAGGACGTGCG	AGGCGGCCGCaC	ACGTCCTCCA	AATAATTA	12	10	-
4	GCGGACATGGAGGACGTGCG	AGGCGGCCGCaC	ACGTCCTCCATGTC	ATACTTAT	12	14	-
5	GGCCGCGGTACTGCACCAGG	GGGCGCGGACATGGAGGACGTGtGCGGCCGCCT	GGTGCAGTACCG	ATAATTAA	33	12	-
5	GGCCGCGGTACTGCACCAGG	GGGCGCGGACATGGAGGACGTGtGCGGaCGCCT	GGTGCAGTACCG	ATAAGATT	33	12	K
5	GGCCGCGGTACTGCACCAGG	ATGGAGGACGTGtGCGGaCGCCT	GGTGCAGTACCG	AATTAAAT	23	12	K
5	GGCCGCGGTACTGCACCAGG	GGCGCGGACATGGAGGACGTGtGCGGaCGCCT	GGTGCAGTACCG	ATTTAATC	32	12	K
5	GGCCGCGGTACTGCACCAGG	TGGGCGCGGACATGGAGGACGTGtGCGGaCGCCT	GGTGCAGTACCG	ATAAGAAC	34	12	K
5	GGCCGCGGTACTGCACCAGG	GCGCGGACATGGAGGACGTGtGCGGaCGCCT	GGTGCAGTACCG	ATTAAATT	31	12	K
6	GCTCGCCGCGGTACTGCACC	GAGGACGTGtGCGGCCGCCTGGT	GCAGTACCGCGG	AATTATCT	23	12	-
6	GCTCGCCGCGGTACTGCACC	GAGGACGTGtGCGGCCGatTGGT	GCAGTACCGCGG	ATTAATAA	23	12	L
6	GCTCGCCGCGGTACTGCACC	ATGGAGGACGTGtGCGGCCGatTGGT	GCAGTACCGCGG	AATATACA	26	12	L
6	GCTCGCCGCGGTACTGCACC	GAGGACGTGtGCGGCCGatTGGT	GCAGTACCGCGGC	AATTATTA	23	13	L
6	GCTCGCCGCGGTACTGCACC	GGACGTGtGCGGCCGatTGGT	GCAGTACCGCGG	AATTAAAT	21	12	L
6	GCTCGCCGCGGTACTGCACC	GAGGACGTctGCGGCCGatTGGT	GCAGTACCGCGG	AACATTAA	23	12	D+L
6	GCTCGCCGCGGTACTGCACC	ATGGAGGACGTctGCGGCCGatTGGT	GCAGTACCGCGG	ATATAAAC	26	12	D+L
6	GCTCGCCGCGGTACTGCACC	GAGGACGTctGCGGCCGatTGGT	GCAGTACCGCGGC	ATATACAC	23	13	D+L
6	GCTCGCCGCGGTACTGCACC	GGACGTctGCGGCCGatTGGT	GCAGTACCGCGG	AACTTATT	21	12	D+L
6	GCTCGCCGCGGTACTGCACC	GGACGTttGCGGCCGatTGGT	GCAGTACCGCGG	AATAACTC	21	12	E+L
4	GCGGACATGGAGGACGTGCG	AatCGGCCGCag	ACGTCCTCCATG	AAATAATA	12	12	D+L
4	GCGGACATGGAGGACGTGCG	AatCGGCCGCag	ACGTCCTCCA	ACATAAAC	12	10	D+L
4	GCGGACATGGAGGACGTGCG	AatCGGCCGCaa	ACGTCCTCCATG	AATATATT	12	12	E+L
4	GCGGACATGGAGGACGTGCG	AatCGGCCGCaa	ACGTCCTCCA	CAAATATA	12	10	E+L

Supplementary Table 2. Information of human fibroblasts used in this study

Sample ID	Genotype	Reported AD onset	Age at sampling	Sex	Supplier
GM23967	APOE ε3/ε3	-	52 yr	Male	Coriell
AG11414	APOE ε3/ε4	Early-onset AD	39 yr	Male	Coriell
AG04402	APOE ε3/ε4	Early-onset AD	47 yr	Male	Coriell
AG05810	APOE ε3/ε4	Late-onset AD	79 yr	Female	Coriell

Supplementary Table 3. Information of primers used in this study

Cloning primer	Sequence (5'-3')
ApoE4_target_FP	ggaggaggtgggttttccagtcacacctcaggtacCGGCTGTCCAAGGAGCTG
ApoE4_target_RP	acagctgccttgtaagtcattggtcttaaaggtacCCTCGGTGCTCTGGCCG
La_addition_FP	ccggcggaagctctg
La_addition_RP	gcagggccagagcc

NGS primer	Sequence (5'-3')
mouse_on_target_FP	ACACTCTTTCCCTACACGACGCTCTTCCGATCTCGGAACTGGAGGAACAACTGACCC
mouse_on_target_RP	GTGACTGGAGTTCAGACGTGTGCTCTTCCGATCTGGCGAGGCGCACCCGC
NIH_on_target_FP	ACACTCTTTCCCTACACGACGCTCTTCCGATCTGCCTGGCTAGAAGCACAAGAGG
NIH_on_target_RP	GTGACTGGAGTTCAGACGTGTGCTCTTCCGATCTCCTTTTCTTTTAAAAAGTGGCTAAGATCTACAGCTG
off_target_site1_FP	ACACTCTTTCCCTACACGACGCTCTTCCGATCTCAGGGACTACTCTCCTGCTC
off_target_site1_RP	GTGACTGGAGTTCAGACGTGTGCTCTTCCGATCTCTGCCCTGTGAGGATGTGG
off_target_site2_FP	ACACTCTTTCCCTACACGACGCTCTTCCGATCTGTGCGGTCAAGGCGTAAG
off_target_site2_RP	GTGACTGGAGTTCAGACGTGTGCTCTTCCGATCTCAAGGTGCAGCCGTGAAC
off_target_site3_FP	ACACTCTTTCCCTACACGACGCTCTTCCGATCTCTGGACTGTGCCTACAGCC
off_target_site3_RP	GTGACTGGAGTTCAGACGTGTGCTCTTCCGATCTCACTGCGATCGCTGCC
off_target_site5_FP	ACACTCTTTCCCTACACGACGCTCTTCCGATCTGCTCATCTGGATTGAAGCCAG
off_target_site5_RP	GTGACTGGAGTTCAGACGTGTGCTCTTCCGATCTGGGACCCACAGTCGAAGC
off_target_site6_FP	ACACTCTTTCCCTACACGACGCTCTTCCGATCTCGCCCAGCGATGCATTTATTTC
off_target_site6_RP	GTGACTGGAGTTCAGACGTGTGCTCTTCCGATCTCATACAGCAGTGCTCAGTGC
off_target_site7_FP	ACACTCTTTCCCTACACGACGCTCTTCCGATCTGGGACAGGTTTGTTACACCACC
off_target_site7_RP	GTGACTGGAGTTCAGACGTGTGCTCTTCCGATCTGGTGGGACTTCATCTTTGCTG
off_target_site8_FP	ACACTCTTTCCCTACACGACGCTCTTCCGATCTGGAAGTCTTCAAAAGGCAACTGG
off_target_site8_RP	GTGACTGGAGTTCAGACGTGTGCTCTTCCGATCTCCGAATAACCATAACTTTCTGTAGAAAACC
off_target_site9_FP	ACACTCTTTCCCTACACGACGCTCTTCCGATCTGCAAGACACTTCCATCTTCGC
off_target_site9_RP	GTGACTGGAGTTCAGACGTGTGCTCTTCCGATCTGGTTTCGTGGTTGGTGGATTG
off_target_site10_FP	ACACTCTTTCCCTACACGACGCTCTTCCGATCTCTCTCTGTCCCGGCTGG
off_target_site10_RP	GTGACTGGAGTTCAGACGTGTGCTCTTCCGATCTCAATTCCTCTGGCCGCG
off_target_site11_FP	ACACTCTTTCCCTACACGACGCTCTTCCGATCTGTAACCTGAGCGGCCG
off_target_site11_RP	GTGACTGGAGTTCAGACGTGTGCTCTTCCGATCTCTGCTTCAGCCCGGAGC
off_target_site12_FP	ACACTCTTTCCCTACACGACGCTCTTCCGATCTCAGGTTGCTACAGTTCTTGAAGAC
off_target_site12_RP	GTGACTGGAGTTCAGACGTGTGCTCTTCCGATCTCAGTCGCCTTTCGCCAC
off_target_site13_FP	ACACTCTTTCCCTACACGACGCTCTTCCGATCTCTCAAAGCCTGGGATCTGAGC
off_target_site13_RP	GTGACTGGAGTTCAGACGTGTGCTCTTCCGATCTCTGGGCAGCACAGTAGAAATGG
off_target_site14_FP	ACACTCTTTCCCTACACGACGCTCTTCCGATCTCCAGTTCAAGTATCAGTGCACG
off_target_site14_RP	GTGACTGGAGTTCAGACGTGTGCTCTTCCGATCTCAGCGCAGCCACACTCTC
off_target_site15_FP	ACACTCTTTCCCTACACGACGCTCTTCCGATCTCGCCAGCGGCTGTCC
off_target_site15_RP	GTGACTGGAGTTCAGACGTGTGCTCTTCCGATCTGGTCAACCTGGAGAAGGAAAACTG
off_target_site16_FP	ACACTCTTTCCCTACACGACGCTCTTCCGATCTGCAGATGAACCCTAAGCCGG
off_target_site16_RP	GTGACTGGAGTTCAGACGTGTGCTCTTCCGATCTCGCCAGCCTCCCGTTC
