# Supplementary material for: Therapeutic Gene Editing of APOE4 in Sporadic Alzheimer's Disease via Prime Editor 7
Source: Adv Sci (Weinh). 2026 Jul 17;13(57):e76658. doi: 10.1002/advs.76658 (PMC13379251; doi:10.1002/advs.76658)
Supplement: Supplementary file 2 [file ADVS-13-e76658-s002.pdf]

Supplementary Figure 1.

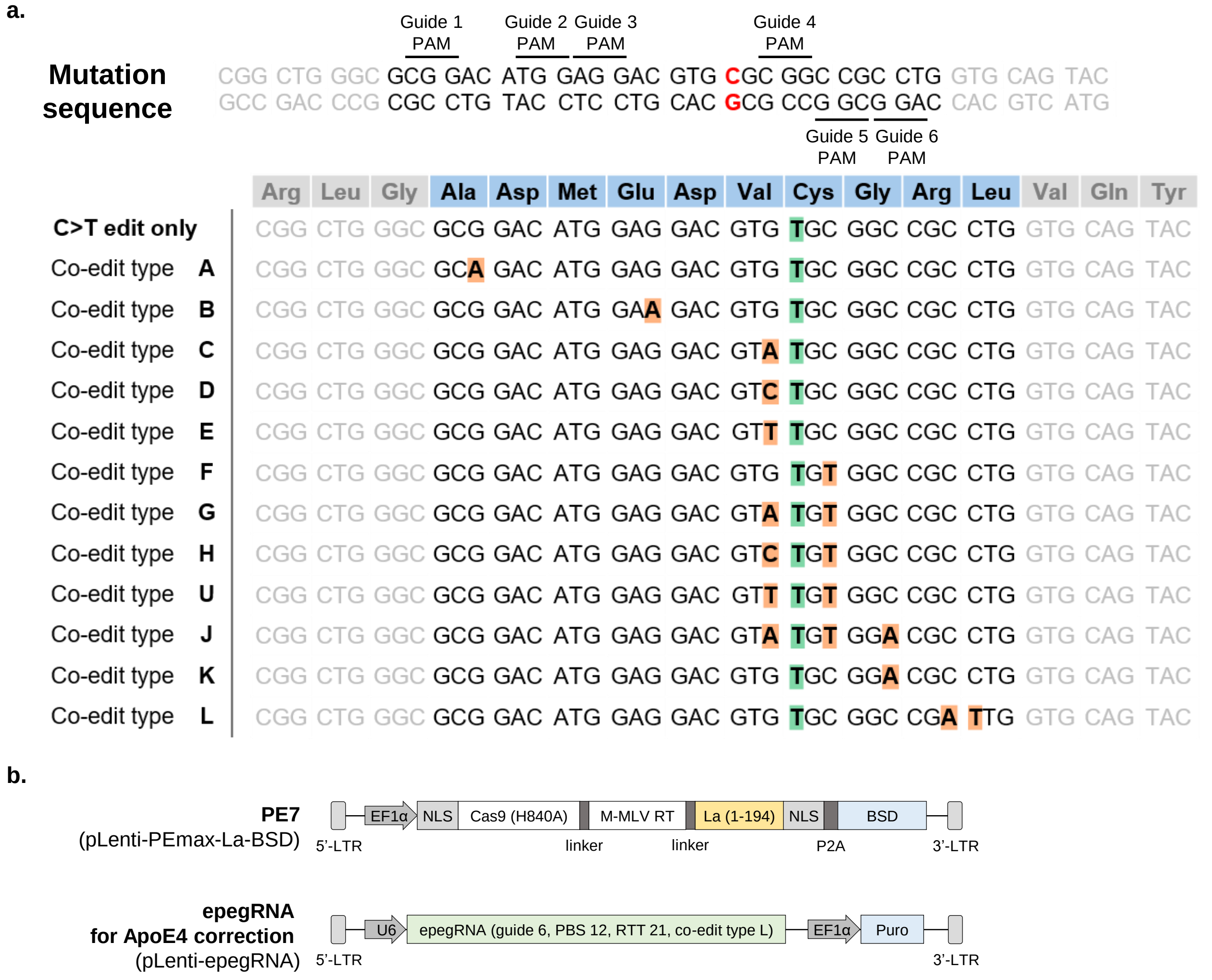

(a) Schematic representation of the pegRNA design. Types of silent edits and the intended edit are indicated in yellow and green, respectively. **(b)** Schematic maps of the lentiviral vectors used in this study. The upper panel shows the PE7-expressing lentiviral construct (pLenti-PEmax-La-BSD), containing the EF1α promoter-driven PE7 cassette composed of Cas9(H840A), M-MLV reverse transcriptase (RT), and the La domain (aa 1–194), followed by a P2A-blasticidin resistance (BSD) cassette. The lower panel shows the APOE-targeting epegRNA lentiviral construct (pLenti-epegRNA), in which the human U6 promoter drives expression of the epegRNA (guide 6, PBS 12 nt, RTT 21 nt, silent co-edit design), together with an EF1α-driven puromycin resistance cassette.

Supplementary Figure 2.

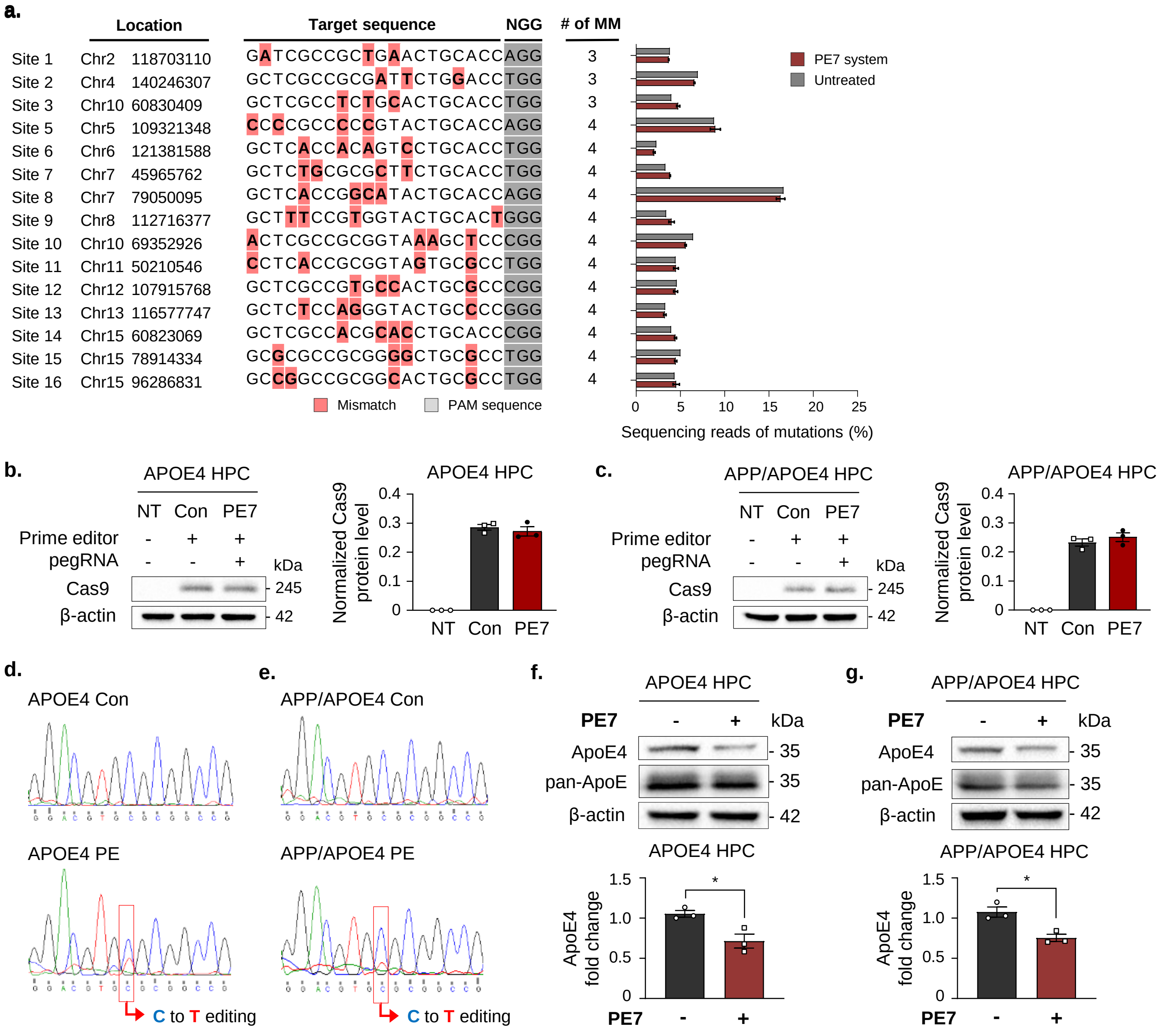

(a) Off-target prime editing evaluation of the selected pegRNA with PE7 system in hippocampal tissue of ApoE4-KI mice following PE7 injection. Left: sequence information for potential off-target sites in the mouse genome. Right: targeted deep sequencing results at these sites. Mutations comprised insertions, deletions, and substitutions. Error bars represent the standard deviation of two replicates. **(b,c)** Western blot analysis of Cas9 expression in hippocampal tissues (HPC) from APOE4-KI mice (b) and APP/APOE4-KI mice (c) following stereotaxic delivery of PE7 lentivirus, with quantification. NT, non-treated; Con, control lentivirus; PE7, PE7 plus pegRNA lentivirus. **(d,e)** Representative Sanger sequencing chromatograms showing the intended C-to-T substitution at the APOE4 target locus in hippocampal tissues from APOE4-KI (d) and APP/APOE4-KI (e) mice following PE7 treatment. **(f,g)** Western blot analysis of ApoE4 and pan-ApoE protein levels in hippocampal tissues from APOE4-KI mice (f) and APP/APOE4-KI mice (g) following PE7 treatment, with quantification of ApoE4 levels. Data are presented as mean ± SEM. Statistical significance was determined by one-way ANOVA with Tukey's multiple comparisons test (b, c) or unpaired two-tailed Student's t-test (e, f). *P < 0.05, **P < 0.01; n = 3 biologically independent samples per group, except (a) (n = 2).

Supplementary Figure 3.

a.

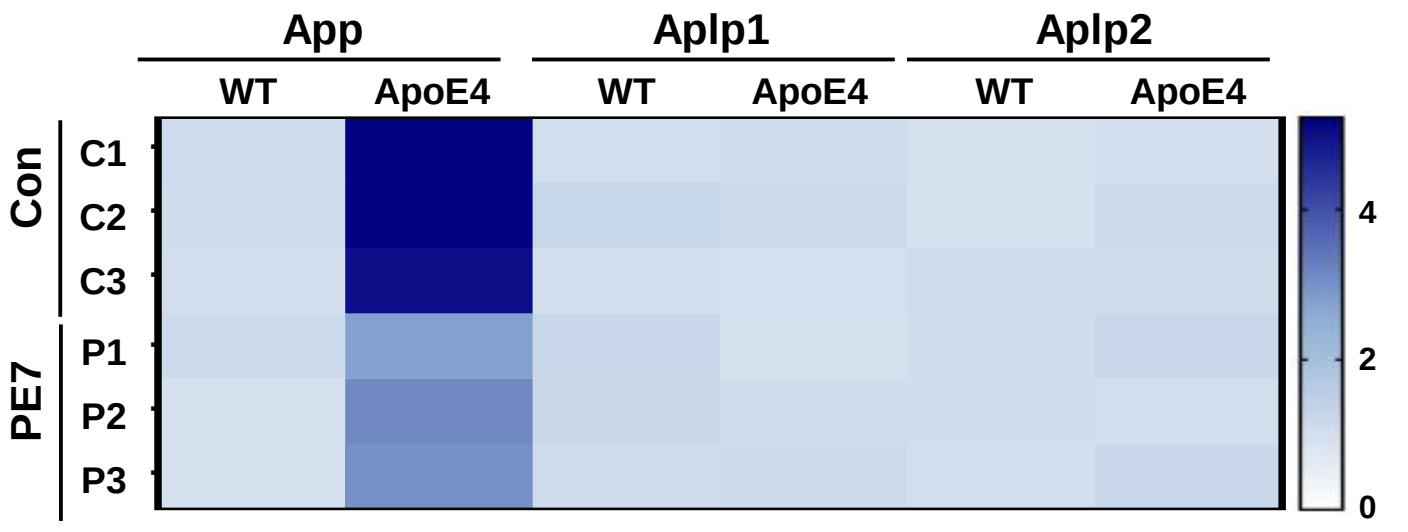

(a) Quantitative RT-PCR analysis of App, Aplp1, and Aplp2 expression in the hippocampus (HPC) of ApoE4 knock-in mice compared to WT controls. Heatmap shows relative mRNA expression levels in control (Con) and PE7-treated (PE7) groups. Data are normalized to internal reference genes and presented as log2 fold-change values. n = 3 biologically independent samples per group.

Supplementary Figure 4.

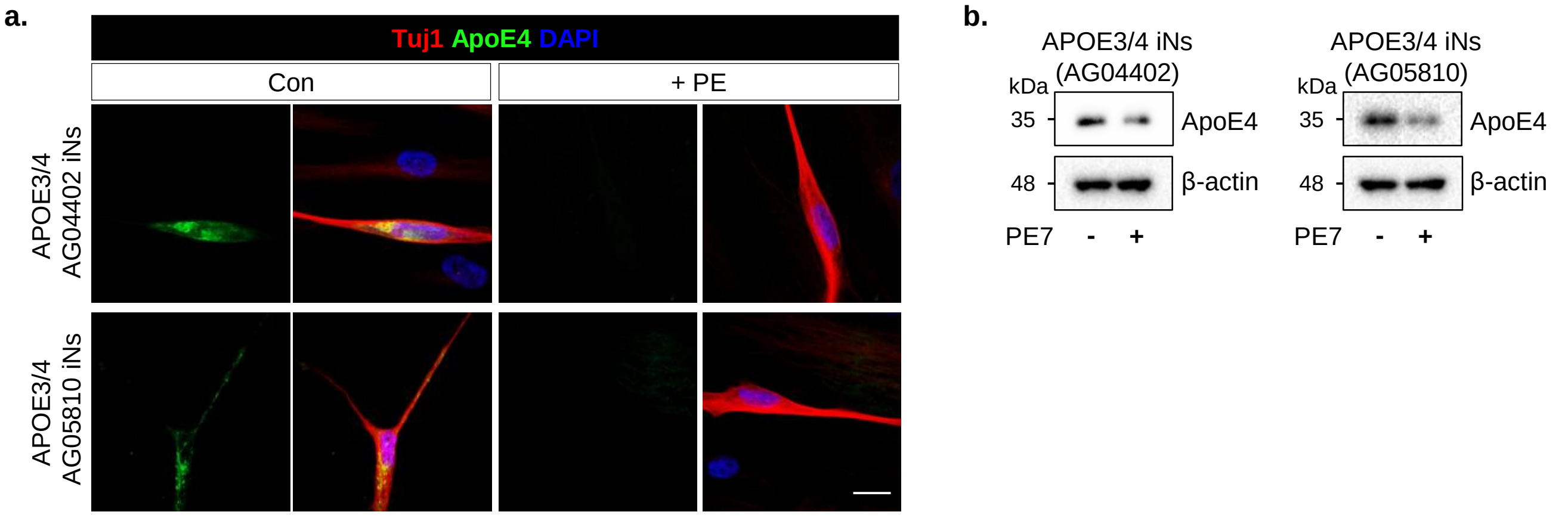

(a) Immunocytochemistry of induced neurons (iNs) derived from AD patient fibroblasts (AG04402 and AG05810), stained for Tuj1 (red), ApoE4 (green), and DAPI (blue), comparing control and PE-treated groups. Scale bar, 20 μm. **(b)** Western blot analysis of human ApoE4 expression in iNs derived from AG04402 and AG05810 fibroblasts, comparing control and PE-treated groups. The corresponding quantification from three biologically independent experiments is shown in Figure 4c.
